# Beyond Benford's Law: Distinguishing Noise from Chaos

**DOI:** 10.1371/journal.pone.0129161

**Published:** 2015-06-01

**Authors:** Qinglei Li, Zuntao Fu, Naiming Yuan

**Affiliations:** 1 Laboratory for Climate and Ocean-Atmosphere Studies, Dept. of Atmospheric and Oceanic Sciences, School of Physics, Peking University, Beijing, China; 2 Department of Geography, Climatology, Climate Dynamics, and Climate Change, Justus-Liebig University Giessen, Giessen, Germany; CNRS, FRANCE

## Abstract

Determinism and randomness are two inherent aspects of all physical processes. Time series from chaotic systems share several features identical with those generated from stochastic processes, which makes them almost undistinguishable. In this paper, a new method based on Benford's law is designed in order to distinguish noise from chaos by only information from the first digit of considered series. By applying this method to discrete data, we confirm that chaotic data indeed can be distinguished from noise data, quantitatively and clearly.

## Introduction

Time series from chaotic systems (CSs) share with those from stochastic processes (SPs) some properties make them almost undistinguishable. Though behind the veil of apparent randomness, many series from CSs are highly ordered [1–3], the distinction between chaotic and stochastic processes is still a long-standing challenge [4–18]. Moreover, experimental chaotic records are unavoidably contaminated with noise, which makes the distinction task even more complicated.

The discrimination between chaotic and stochastic processes has drawn much attention, since irregular and apparently unpredictable behaviors are often observed in natural measurements. Many studies have been done aim to uncover the cause of unpredictability governing these systems, and much effort has been further devoted in understanding this topic [4–18]. First of all, exponential power-spectra have been identified in many idealized nonlinear systems, and are taken to be characteristics of low-dimensional chaos to differentiate chaos from stochastic processes, whose power-spectra show power-law behavior [4–7]. Nonlinear forecasting [8,9] has also been applied to make tentative distinctions between dynamical chaos and measurement errors, since the accuracy of nonlinear forecast diminishes with increasing prediction time intervals for chaotic series, but for stochastic series, it does not. Recently, network and symbolic dynamics related methods [10–18] are used to handle this issue, where structural information among consecutive points in physical or phase space are used to characterize and distinguish stochastic from chaotic processes.

Although above mentioned methods have been successfully applied to distinguish stochastic from chaotic processes, the authors of each method have only explored the related magnitude or permutation information of the analyzed processes, such as power-spectrum method or network based methods. We note that digital information has never been used so far to characterize and further distinguish stochastic from chaotic processes. Actually, digital information is of great importance to characterize specific process. For example, the first digits in many datasets are not uniformly distributed as expected, but heavily skewed toward the smaller digits. This phenomenon was first found by Simon Newcomb in 1881 [19]. Nobody showed interests in this discovery, until 1938 when Frank Albert Benford [20] investigated some 20 tables of 20229 numbers and drawn the conclusion that the first digit probability distribution in many data sets is

$$P_{B}\left(d\right)={\text{log}}_{10}\left(1+1/d\right)$$ (1)

where *d* = 1,2,…,9 is the first digit. It was named as Benford's Law (BL) later by the scientific community. Many scientists in different fields have tried to explain the underlying reasons for BL [20–26], but a successful explanation remains elusive [27,28]. However. although there is no accepted interpretation, BL is nearly taken as an universal law. In recent years, most BL related studies are limited in validating whether particular datasets follow this law [29,30], detecting frauds in election and accounting [31,32], as well as testing physical system transition [33,34]. Especially, Tolle and his coauthors [35] examined three low-dimensional chaotic models of dynamical systems, and found examples of either compliance with or deviance from Benford's law, which depends upon the models and the parameters.

Can Benford's law be explored to characterize and distinguish stochastic from chaotic processes? The answer from the Toll's results is no. However, the observed dynamics may be strongly affected by the resolution scales used to document the behaviors of considered processes [36]. In order to characterize complex multi-scaled series, it is of fundamental importance to incorporate the multiple scale in devising measures [36]. Costa et al [37]. and Zunino et al. [13] have introduced multi-scale entropy (MSE) and multi-scale permutation entropy (MPE) to successfully distinguish different states of analyzed processes or dynamical systems, respectively. These results show the importance of multi-scale in characterizing the analyzed processes or systems. Here for the first time we introduce the multi-scale to Benford's law analysis, and the results show that it does help us in distinguishing chaos from noise.

## Materials and Methods

### Generating SPs

We generate three kinds of well-known stochastic processes by Fourier transform technique: (1) Noise with *f* ^-*k*^ power spectra, (2) Fractional Gaussian noise (FGN) and (3) Fractional Brownian motion (FBM). All three SPs are a particular class of colored noise which represent stochastic (infinite-dimensional) systems with different power-law spectra [13,14].

### Noise with *f* ^-*k*^ power spectra

1. Generate a set {*u*_*i*_,*i* = 1,2,…,*N*} of independent Gaussian variables of zero mean and variance one, and compute the discrete Fourier transform of the sequence $\left\{{\hat{u}}_{k}^{1}\right\}$.
2. Correlations are incorporated in the sequence by multiplying the new set by the desired spectral density *f* ^-*k*^, yielding $\left\{{\hat{u}}_{k}^{2}\right\}$;
3. Now,$\left\{{\hat{u}}_{k}^{2}\right\}$ is symmetrized so as to obtain a real function and then the pertinent inverse Fourier transform {*x*_*i*_} is obtained, after discarding the small imaginary components produced by our numerical approximations.

### Fractional Gaussian noise (FGN) and Fractional Brownian motion (FBM)

FBM is the only family of processes which is (a) Gaussian, (b) self-similar, and (c) endowed with stationary increments [14,38,39]. The normalized family of these Gaussian processes, {*B* ^*H*^(*t*),*t*>0}, is endowed with these properties: (i) *B* ^*H*^(0) = 0 with probability 1, (ii) *E*[*B* ^*H*^(*t*)] = 0 (zero mean), and (iii) covariance given by

$$E\left[B^{H}\left(t\right)B^{H}\left(s\right)\right]=\left(t^{2H}+s^{2H}-{\left|t-s\right|}^{2H}\right)/2$$

for *t*,*s*∈*R*. Here *E*[] refers to the average computed with a Gaussian PDF. The power exponent 0<*H*<1 is commonly known as the Hurst parameter (exponent). These processes exhibit ‘‘memory” for any Hurst parameter except for *H* = 1/2, as one realizes from Eq (11). The case *H* = 1/2 corresponds to classical Brownian motion and successive motion increments are as likely to have the same sign as the opposite (there is no correlation among them). Thus, Hurst’s parameter defines two distinct regions in the interval (0,1). When *H*>1/2, consecutive increments tend to have the same sign so that these processes are persistent. For *H*<1/2, on the other hand, consecutive increments are more likely to have opposite signs, and we say that they are anti-persistent. Let us introduce the quantity Fractional Gaussian noise (FGN) as the FBM increments, 2*W* ^*H*^(*t*) = *B* ^*H*^(*t*+1)-*B*(*t*)^*H*^, so as to express our Gaussian noise in the fashion

$$\rho \left(k\right)=E\left[W^{H}\left(t\right)W^{H}\left(t+k\right)]=[{\left(k+1\right)}^{2H}-2k^{2H}-{\left|k-1\right|}^{2H}\right]/2,k>0$$

Note that for *H* = 1/2 all correlations at nonzero lags vanish and {*W* ^1/2^(*t*),*t*>0} thus it represents white noise. The FBM and FGN processes are continuous but non-differentiable processes (in the classical sense). It is possible to define a generalized power spectrum of the form: Φ∝|*f*|^-*β*^, with *β* = 2*H*+1,1<*β*<3 for FBM and *β* = 2*H*-1,-1<*β*<3 for FGN. For evaluating the FBM and FGN time series, here we use a modified Fourier filtering technique [39,40], which is both exact and fast.

### Generating CS

In order to compare results given in our proposed method with those from other methods, all the CSs chosen in this paper are those used to distinguish noise from chaos in the literature [13–17, 41].

### Noninvertible chaotic maps

(1) Gauss map:xn+1=1xn (Mod 1). (2) Linear congruential generator: *x*_*n*+1_ = *ax*_*n*_+*b* (Mod 1c),where *a* = 7141,*b* = 54773,*c* = 259200. (3) Schuster map: $x_{n+1}=x_{n}+x_{n}^{z}\;(\text{Mod 1})$ in the fully chaotic region, where *z* = 3/2.

### Dissipative chaotic maps

(4) Dissipative standard map:{xn+1=xn+yn+1 (Mod 2π)yn+1=bxn+ksin(xn) (Mod 2π),where *b* = 0.1,*k* = 8.8. (5) Kaplan Yorke map:{xn+1=2xn (Mod 1)yn+1=αyn+cos(4πxn), in the fully chaotic region, where *α* = 0.2. (6) Sinai map:{xn+1=xn+yn+δsin(2πyn) (Mod 1)yn+1=xn+1+2yn (Mod 1), where *δ* = 0.1. (7) Tinkerbell map:{xn+1=xn2-yn2+axn+bynyn+1=2xnyn+cxn+dyn, where *a* = 0.9,*b* = -0.6013,*c* = 2.0,*d* = 0.50

(8) Chirikov standard map:{xn+1=xn+ksin(yn) (Mod 2π)yn+1=xn+1+yn (Mod 2π) with *k* = 0.2. (9)Lorenz three-dimensional chaotic system: {x˙=ay-axy˙=bx-y-xzz˙=xy-cz in the fully chaotic region with *a* = 10,*b* = 25,*c* = 8/3.

Here we also analyze more other deterministic time series generated by chaotic maps (see more details in S1 File), Details for these maps can be found in the references [14–16,41]. Even though the presented list of chaotic maps is not exhaustive, it could be taken as representative of common chaotic systems [41]. For all the CS cases, 10 time series with 10^5^ data points each were analyzed, and each series starting at a different initial conditions.

## Results and Discussions

### The first digit distribution at different scales for CSs and SPs

After generating the chaotic and stochastic time series *x*_*i*_, *i* = 1,2,3…*N* (where *N* is the length of analyzed series) by using simple schemes described above (see Materials and Methods), We define *P*_*s*_(*d*),*d* = 1,2,…,9 as the frequency of the first non-zero digit in the consecutive coarse-grained time series:

$$y_{j}^{s}=1/s\sum_{(j-1)s+1}^{js}x_{i},\;1\leq j\leq \left[N/s\right]$$ (2)

which is determined by the scale factor *s* = 1,2,3…,20 [37].

**Fig 1:**
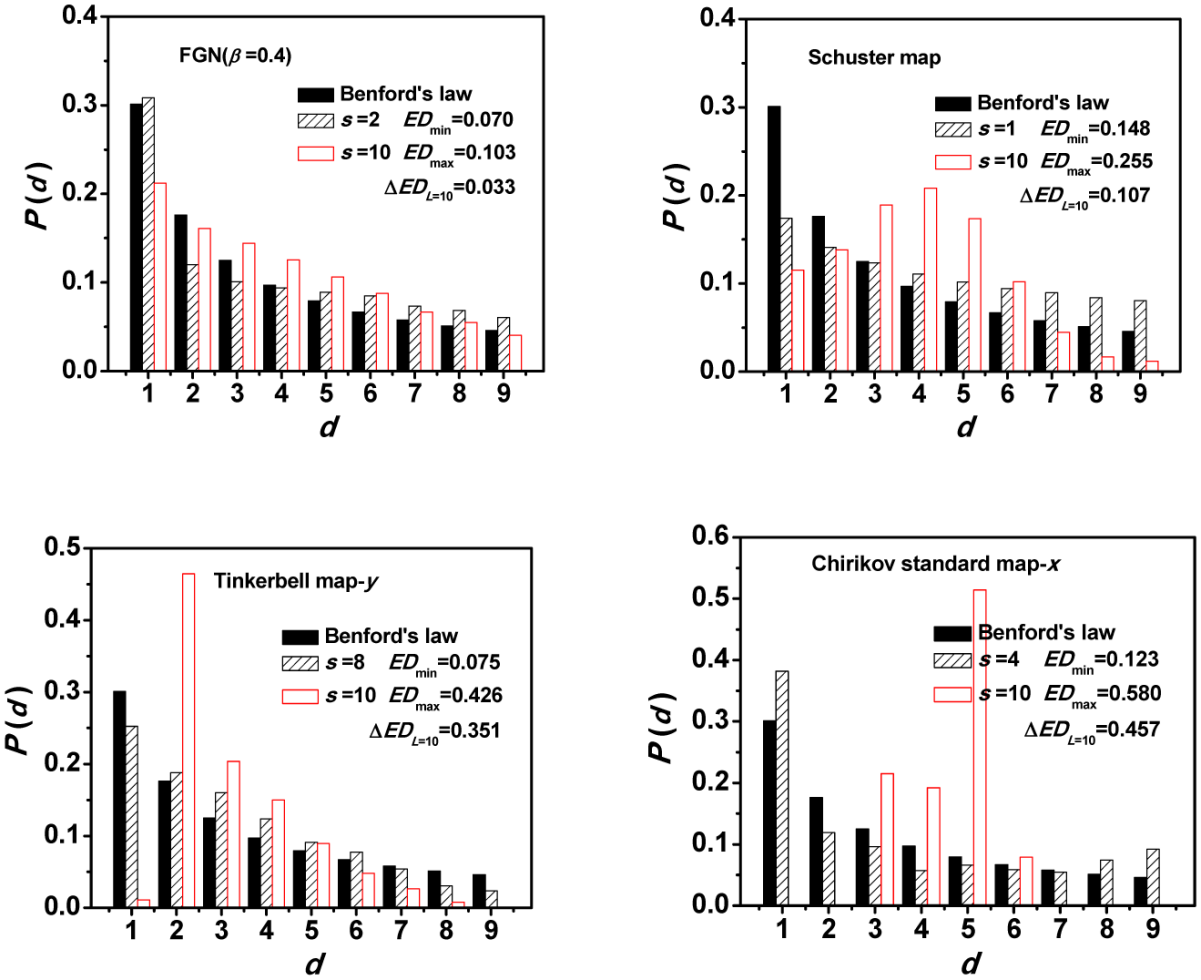
Fourtypical examples of the first digit distribution for time series at different scales. The stochastic process: FGN *β* = 0.4,the noninvertible chaotic maps: Linear congruential generator, the dissipative chaotic maps: Tinkerbell map-*y* and the conservative chaotic maps: Chirikov standard map-*x*.

The frequency with which different first digits occur is shown to be differently sensitive to scales for the different kinds of processes. Fig 1 plots the first digit distributions at two predominant scales from three kinds of chaotic processes and one kind of stochastic process, with the theoretical Benford's Law (Eq (1)) shown for comparison. Time series derived from each process has identical length of 10^5^ points. The two typical scales are chosen according to the conformance degree between the first digit distribution of each process and the Benford's law. One is the best compliance with and the other is the greatest deviance from Benford's law. We find that time series derived from chaotic and stochastic processes have different characteristics. For stochastic process, differences from the theoretical Benford's law are much smaller at both the compliance scale and deviance scale (see Fig 1 for FGN with *β* = 0.4), and the fundamental feature is still heavily skewed toward the smaller digits. Moreover, all the stochastic processes take this similar behavior (see Figure A in S1 File). However, differences for all three kinds of chaotic processes (see Fig 1 for the noninvertible chaotic maps: Linear congruential generator, the dissipative chaotic maps: Tinkerbell map-*y* and the conservative chaotic maps: Chirikov standard map-*x*) are significant. At the deviance scale, the frequency distributions of the first significant digits are not the expected frequency distribution from Benford's law, whereas some digits are missing and only specific digit(s) exist, which indicates a drastic breakdown of Benford's law. Actually, the drastic breakdown of Benford's law can be found in all the chaotic systems we have analyzed (Figures B–D in S1 File).

### Euclidean distance for CSs and SPs

From the frequency distribution of the first digits at different scales, we just find the qualitative distinction between the chaotic and stochastic processes. The difference between the frequency distributions of the first significant digits of the considered processes and the expected frequency distribution from Benford's law can be quantified by a parameter. Here we choose this parameter as Euclidean distance, and it is defined as

$$ED\left(s\right)=\sqrt{\sum_{d=1}^{9}{\left[P_{s}\left(d\right)-P_{B}\left(d\right)\right]}^{2}}$$ (3)

According to this definition and the Benford's law, the maximum value of *ED* is 1.036 for the case when time series has only one specific digit 9 appears, but other eight digits are forbidden; the minimum value of *ED* is 0 for time series fully following the Benford's law distribution.

**Fig 2:**
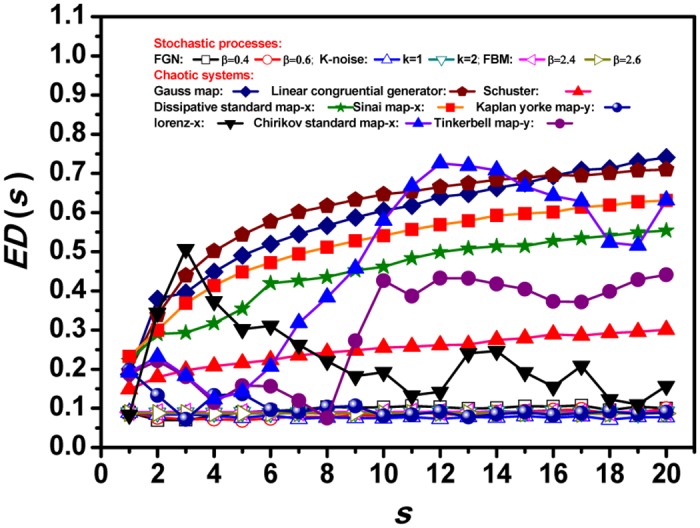
The changing values of *ED*(*s*) with scale factors for different SPs and CSs. It can be taken as a sensitive measure of the underlying dynamics.

Quantitatively, the values of *ED*(*s*) do not change much, from 0.070 to 0.103, for stochastic process, see Fig 1a, but there are substantive changes for the chaotic processes. As shown in Fig 1b–1d, *ED* value changes from 0.148 to 0.255 for Schuster map, from 0.075 to 0.426 for Tinkerbell map-*y*, from 0.123 to 0.580 for Chirikov standard map-*x*. Therefore, the changing values of *ED*(*s*) with scale factor*s* seem to be a sensitive measure of the underlying dynamics. In Fig 2, details on how the *ED*(*s*) changes with scale factor*s* are shown. First of all, we can see that at some scales, we can't distinguish CSs from SPs since many of CSs have the same lower *ED* values as SPs. This is consistent with the conclusion there are CS examples, which are both compliance with and deviance from Benford's law, depending upon the chaotic models and the parameters [35]. Secondly, with increasing scale factor, the *ED* remains almost unchanged for all stochastic process (see Figure E in S1 File), but varies significantly for all deterministic chaotic systems (see Figures E–H in S1 File). The differences are not difficult to understand since the CSs are multi-scaled and have a finite dimensional attractor in phase space, while the SPs do not [41]. Thirdly, different chaotic systems have their own largest *ED* values at the different specific deviance scale, because they are distinctively multi-scaled and have different dimensions [41].

### Distinguishing index Δ*ED* between CSs and SPs

**Fig 3:**
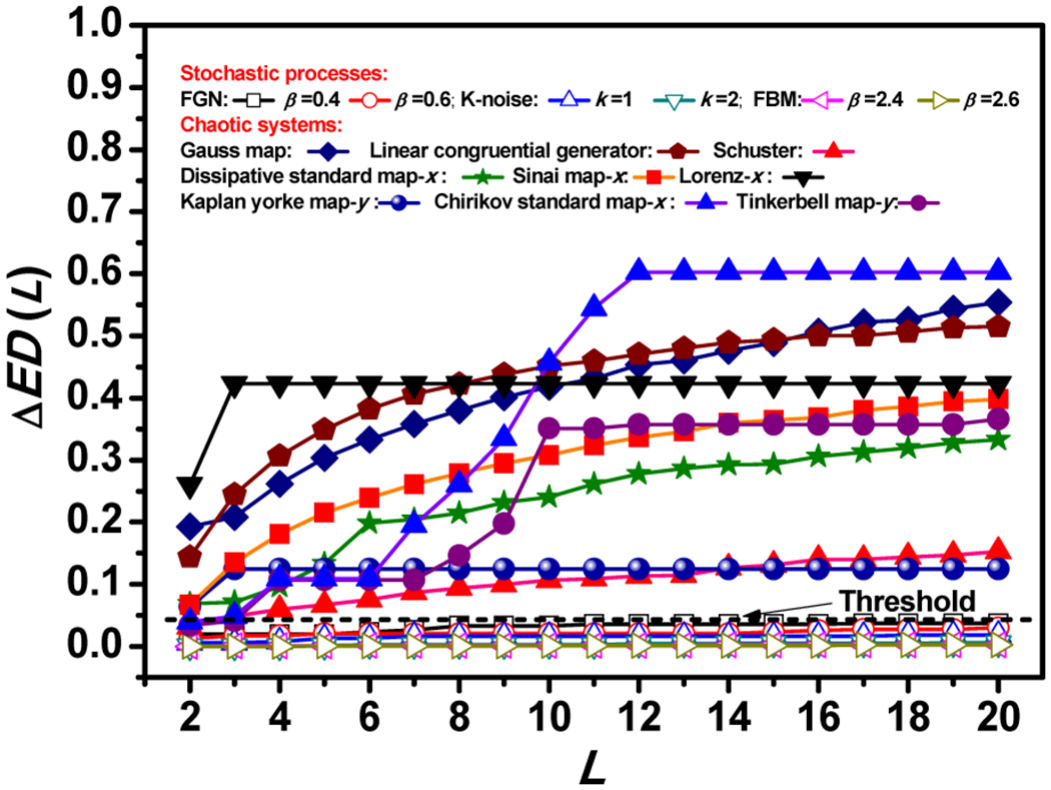
The results of Δ*ED*(*L*) for different SPs and CSs. It is almost unchanged near zero for SPs, but changing a lot for CSs. The distinction between CSs and SPs is significant when the scale range is larger than 4. A threshold of 0.039 (dash line) chosen as the maximum of Δ*ED*(*L*) from 1000 SPs is shown in the figure.

Since the *ED* is nearly invariant for SPs but changes greatly for CSs, in order to clearly display the quantitative differences between CSs and SPs, we define Δ*ED* as the *ED* variation range within given scale ranges:

ΔED(L)=max{ED(s), s∈[1,L]}−min{ED(s), s∈[1,L]} (4)

where max{*ED*(*s*),sϵ[1,*L*]} and min{*ED*(*s*),*s*ϵ[1,*L*]} are the maximum and minimum value of *ED*(*s*) within the scale range [1,*L*], respectively. Fig 3 shows that Δ*ED* is almost invariant close to zero for SPs with the varying scale range *L*, but for CSs it departs with increasing *L* from a nonzero threshold, which is determined as the maximum of Δ*ED* calculated from 1000 SPs at each given range scale, (Figures I–K in S1 File). We can see that the distinction between CSs and SPs increases significantly as the scale range *L* increases. When the scale range larger than 4, all the values of Δ*ED* from CSs are above the threshold and all those from the SPs are below the threshold. Fig 3 also shows us the Δ*ED* gap between CSs and SPs broadens as the scale range increases, which help us make distinction easier. What’s more, the results show us that Δ*ED* can also help us distinguish different CSs at large scale range.

## Conclusions

Motivated by BL, here we exploit the first digit distribution to distinguish different chaotic processes from stochastic processes. The Euclidean distance is adopted to quantify the differences between the first digit distributions from different processes and the theoretical BL. In order to find the difference between the chaotic and stochastic processes, it is of fundamental importance to explicitly incorporate the concept of multiple scales to characterize complex multi-scaled signals. Here with scale factor increasing, we find the changing *ED*(*s*) for CSs and nearly invariant *ED*(*s*) for SPs are basically predominated qualitative difference between the chaotic processes and the stochastic ones. Actually, this kind of difference between the chaotic processes and the stochastic ones is insensitive to the chosen measures used to quantify the differences between the first digit distributions from different processes and the theoretical BL, and the conclusion is robust to different chosen measures [42,43], see details in Figure L in S1 File.

From the above qualitative difference between the chaotic processes and the stochastic ones, we further define a quantitative index, Δ*ED*(*L*) to quantify the difference between the chaotic processes and the stochastic ones. Compared with other strategies [4–17] given to distinguish the chaotic processes from the stochastic ones, there are some advantages deserve to mention.

First of all, our strategy only makes use of the first digit information of considered series, which doesn’t require high precision recording as required in other amplitude or phase based methods.

Secondly, it is an objective index, and there are no subjective choices in this measure calculation. Selecting the first digit is a way to coarse grain the time series and also a way to develop a symbolic dynamic system. The crucial difference between the current approach and the symbolic method propose in reference [13] is that we symbolize the time series by the first digits but the reported multi-scale permutation entropy approach [13] symbolize the time series through comparing the neighbor values in specifically dimensional ordinal pattern. Although a successful explanation of BL has remained elusive [22–26], some basic properties are clear for Benford's law. It has been proven that BL represents the only probability distribution which is both scale and base invariant [27,28]. The scale invariance of BL means that if first digits of the variable *x* follow (1), then so will the first digits of the rescaled variable *λx* for any nonzero value of *λ*. The base invariance means that BL is independent of the base *d* we used. In the binary base (*d* = 2), octal base (*d* = 8), or other base systems, the data, as well as in the decimal system (*d* = 10), all follow the general first digit law:

$$P_{k}={\text{log}}_{d}\left(1+1/k\right);\;k=1,2,\cdots ,d-1$$ (5)

So, the choice of *d* = 10 doesn’t affect the objectiveness of our method.

Thirdly, Since the Benford's law is not derived from stationary processes, so the Benford's distribution analysis doesn’t require the stationary condition of considered series. This will make the method introduced in this paper more applicable than other methods reported in the literature, where the basic assumption is the stationary condition of the considered processes.

Fourthly, the quantifier proposed in this paper is quantitative, whereas most of other methods reported in the literature are qualitative. Here we give the strict threshold to distinguish the chaotic processes from the stochastic ones, but other methods don’t give their own threshold.

Fifthly, since we only make use of the first digit information of the analyzed series, the algorithm of our method is simple and rapidly calculated.

Sixthly, this method works for series of short data length, and a minimum data length of 4000 is enough to distinguish most of chaotic processes from stochastic ones clearly (Figure M in S1 File), whereas other methods require the length of time series is much longer.

The last one is that our method works better than other qualitative methods, for example, there are always some chaotic maps, which are located nearby the noise “frontier” in some qualitative qualifiers [13–16], and this makes the distinction between these chaotic maps and stochastic processes not so clearly and fully. These chaotic maps are the dissipative standard map, Sinai map, Arnold’s cat map, Gauss map, logistic map and linear congruential generator. However, we can see all these considered chaotic maps can be quantitatively distinguished fully and clearly from the stochastic processes just at the scale range *L* = 4 and only with data length of 4000, see details in Fig 3.

In order to check the robustness of the above results, we also investigated other processes like an Ornstein-Uhlenbeck process [44–47] combining a ‘deterministic’ term and a stochastic term, all ΔED calculated from Ornstein-Uhlenbeck process locates below the threshold which is consistent with the results of stochastic processes, regardless the chosen values of parameters in the Ornstein-Uhlenbeck process (Figure N in S1 File). Even for some high-dimensional chaotic systems, if not all, the conclusions given above are still robust. For example, the high-dimensional chaotic Mackey-Glass system [48–50], ΔED can be applied to distinguish it from the stochastic processes easily (Figure O in S1 File).

At last, it should be noted that recorded series are always contaminated with observational noise. The proposed quantifiers should be robust to these unavoidable noises. We consider series of the following form {*S*_*i*_,*i* = 1,2,…,*N*}

$$S_{i}=x_{i}+A\varepsilon_{i};\;i=1,2,\cdots ,N$$ (6)

where {*x*_*i*_,*i* = 1,2,…,*N*} and {*ε*_*i*_,*i* = 1,2,…,*N*} are from chaotic processes and white noise, respectively. Here *A* can be taken as the amplitude ratio between the additive noise and the chaotic processes. We consider series with data points 10^5^ and amplitude ratio in the range 0≤*A*≤1.

**Fig 4:**
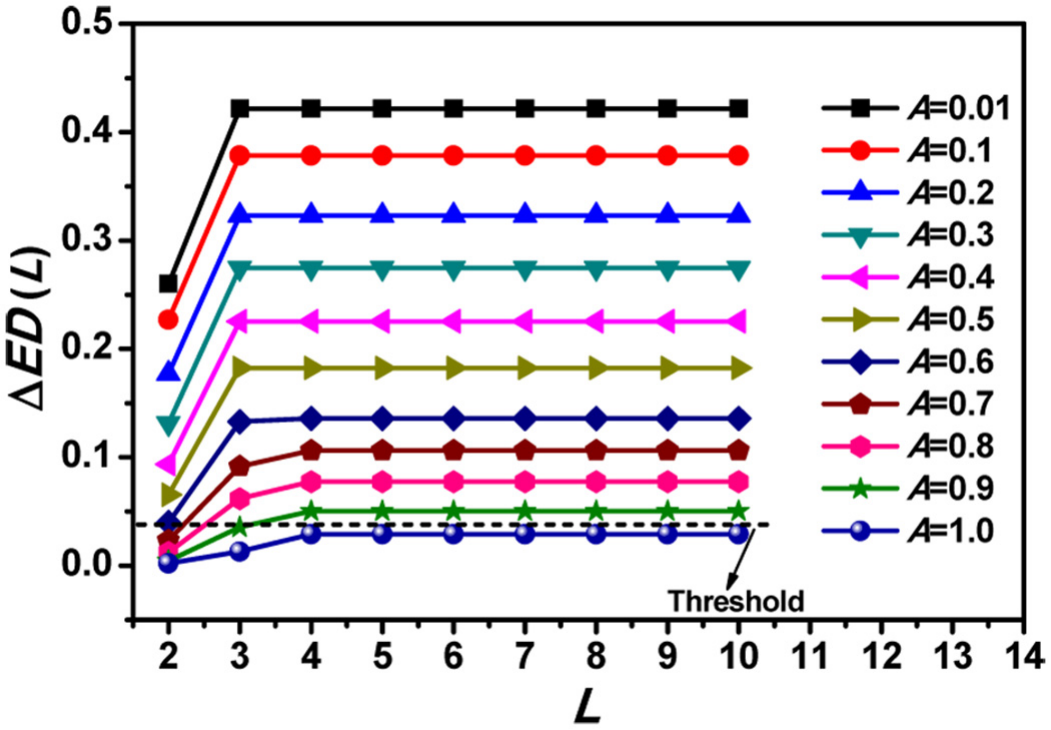
The results of *ED*(*s*) and Δ*ED*(*L*) for CSs contaminated with noise, taking Lorenz-*x* as an example, here we consider series with data points 10^5^ and amplitude ratio in the range 0≤*A*≤1.

Just as expected, the qualifier Δ*ED*(*L*) is robust to the contaminated observational noise when the amplitude ratio *A* is smaller, see Fig 4. This is because only the first digit information of data has been taken into account in this qualifier, when the amplitude ratio *A* is small, the contaminated observational noise can just affect the latter digits from the chaotic processes but not the leading digits. As the amplitude ratio *A* increases, more noise information can enter into the first digit statistics from the chaotic processes, the distinction between contaminated chaotic process and the noise will become difficult and even impossible, see the case for *A* = 1. These conclusions are also robust to the noise of different colors when the amplitude ratio A is smaller (Figure P in S1 File), as the amplitude ratio A increases, the distinction between contaminated chaotic process and the noise will become difficult and even impossible with given finite range, such as *L = 6* for the case for *A = 0*.*9*. However, if larger range *L* is chosen, of course the data length will be extended, we still can distinguish chaos from noise even the sequence from chaotic systems contaminated colored noise. More complicated cases will be met when internal noise is considered, since the internal noise can alter the dynamical behaviors when the internal noise dominates the considered system. So the qualifier ΔED is only robust to the contaminated internal noise when the amplitude ratio *A* is smaller than certain threshold (Figure Q in S1 File).

Due to the above mentioned advantages of our quantifier over others, it may have potential applications to study a wide variety of complex systems, such as complex physiologic dynamics [37] and so on.

## Supporting Information

S1 File Results for more chaotic and stochastic processes. The first digit distribution at different scales for four stochastic time series (**Figure A**), for four noninversative chaotic maps (**Figure B**), for four dissipative chaotic maps (**Figure C**) and for four conservative chaotic maps (**Figure D**). The changing *ED*(*s*) with scale factor s for three kinds of stochastic processes (**Figure E**), for five noninversative chaotic maps (**Figure F**), for nine dissipative chaotic maps (**Figure G**) and for four conservative chaotic maps (**Figure H**). The results of *ED*(*L*) for five noninversative chaotic maps (**Figure I**), for nine dissipative chaotic maps (**Figure J**) and for four conservative chaotic maps (**Figure K**) comparing with those from stochastic processes. The comparative results for different quantifiers (**Figure L**). The Δ*ED*(*L* = 10) versus different data lengths for chosen CSs and SPs (**Figure M**). The Δ*ED* results for Ornstein-Uhlenbeck process (**Figure N**), for Mackey-Glass system (**Figure O**), for Lorenz system with colored noise FGN (*β* = 0.6) (**Figure P**) and for Lorenz system with stochastic forcing (**Figure Q**).
(DOCX)
