# Supplementary material for: Beyond Benford's Law: Distinguishing Noise from Chaos
Source: PLoS One. 2015 Jun 1;10(6):e0129161. doi: 10.1371/journal.pone.0129161 (PMC4452586; doi:10.1371/journal.pone.0129161)
Supplement: S1 File — The first digit distribution at different scales for four stochastic time series (Figure A), for four noninversative chaotic maps (Figure B), for four dissipative chaotic maps (Figure C) and for four conservative chaotic maps (Figure D). The changing ED(s) with scale factor s for three kinds of stochastic processes (Figure E), for five noninversative chaotic maps (Figure F), for nine dissipative chaotic maps (Figure G) and for four conservative chaotic maps (Figure H). The results of ED(L) for five noninversative chaotic maps (Figure I), for nine dissipative chaotic maps (Figure J) and for four conservative chaotic maps (Figure K) comparing with those from stochastic processes. The comparative results for different quantifiers (Figure L). The ΔED(L = 10) versus different data lengths for chosen CSs and SPs (Figure M). The ΔED results for Ornstein-Uhlenbeck process (Figure N), for Mackey-Glass system (Figure O), for Lorenz system with colored noise FGN (β = 0.6) (Figure P) and for Lorenz system with stochastic forcing (Figure Q). (DOCX) [file pone.0129161.s001.docx]

**Beyond Benford’s Law: Distinguishing Noise from Chaos**

Qinglei Li^1^ Zuntao Fu^1*^ Naiming Yuan^1,2*^

**1** Laboratory for Climate and Ocean-Atmosphere Studies, Dept. of Atmospheric and Oceanic Sciences, School of Physics, Peking University, Beijing, China.

**2** Department of Geography, Climatology, Climate Dynamics, and Climate Change, Justus-Liebig University Giessen, D-35390, Giessen, Germany.

***** Corresponding author

Email: fuzt@pku.edu.cn (ZTF); naimingyuan@hotmail.com (NMY).

Three kinds of chaotic processes have been analyzed in this paper, and the details related these processes are listed below. The five noninvertible maps are as follows:

(1) Gauss map:

$$x_{n+1}=\frac{1}{x}_{n} (\text{Mod 1})$$

(2) Linear congruential generator:

$$x_{n+1}=ax_{n}+b (\text{Mod 1c})$$

where $a=7141,b=54773,c=259200$.

(3) Schuster map:

$$x_{n+1}=x_{n}+x_{n}^{z} (\text{Mod 1})$$

in the fully chaotic region, where $z=3/2$.

(4) Logistic map:

$$x_{n+1}=rx_{n}(1-x_{n})$$

in the fully chaotic region, where $r=4$.

(5) Skew tent map:

$$x_{n+1}=\left\{\begin{matrix} x_{n}/w,\text{for }0\leq x<w\text{ } \\ \frac{(1-x_{n})}{(1-w)},\text{for }w<x<1 \end{matrix}\right.$$

in the fully chaotic region, where $w=0.1847$.

The ten dissipative maps as follows are all in the fully chaotic region:

1. Delayed logistic map:

$$\left\{\begin{matrix} x_{n+1}=y_{n} \\ y_{n+1}=ay_{n}(1-x_{n}) \end{matrix}\right.$$

where $a=2.27$

1. Dissipative standard map:

$$\left\{\begin{matrix} x_{n+1}=x_{n}+y_{n+1} (\text{Mod 2}\pi) \\ y_{n+1}=bx_{n}+k\sin(x_{n}) (\text{Mod 2}\pi) \end{matrix}\right.$$

where $b=0.1,k=8.8$.

1. Henon map:

$\left\{\begin{matrix} x_{n+1}=1-ax_{n}^{2}+x_{n} \\ y_{n+1}=bx_{n} \end{matrix}\right.$,

where $a=1.4$ and $b=0.3$

1. Holmes cubic map:

$$\left\{\begin{matrix} x_{n+1}=y_{n} \\ y_{n+1}=-ax_{n}+bx_{n}-y_{n}^{3} \end{matrix}\right.$$

where $a=0.2,b=2.77$.

1. Ikeda map:

$$\left\{\begin{matrix} x_{n+1}=1+u(x_{n}\cos t_{n}-y_{n}\sin t_{n}) \\ y_{n+1}=u(x_{n}\sin t_{n}+y_{n}\cos t_{n}) \end{matrix}\right.$$

$u=0.9,t_{n}=0.4-\frac{6}{(1+x_{n}^{2}+y_{n}^{2})}$.

1. Kaplan Yorke map:

$\left\{\begin{matrix} x_{n+1}=2x_{n} (\text{Mod }1) \\ y_{n+1}=\alpha y_{n}+\cos(4\pi x_{n}) \end{matrix}\right.$,

in the fully chaotic region, where $\alpha=0.2$.

1. Lozi map:

$$\left\{\begin{matrix} x_{n+1}=1+y_{n}-a\left|x_{n}\right| \\ y_{n+1}=bx_{n} \end{matrix}\right.$$

with $a=1.7,b=0.5.$

1. Sinai map:

$$\left\{\begin{matrix} x_{n+1}=x_{n}+y_{n}+\delta\sin(2\pi y_{n}) (\text{Mod }1) \\ y_{n+1}=x_{n+1}+2y_{n} (\text{Mod }1) \end{matrix}\right.$$

where $\delta=0.1$.

1. Tinkerbell map:

$\left\{\begin{matrix} x_{n+1}=x_{n}^{2}-y_{n}^{2}+ax_{n}+by_{n} \\ y_{n+1}=2x_{n}y_{n}+cx_{n}+dy_{n} \end{matrix}\right.$,

where$a=0.9,b=-0.6013,c=2.0,d=0.50$.

1. Lorenz three-dimensional chaotic map::

,

in the fully chaotic region with $a=10,b=25,c=8/3$.

The three conservative maps as follows are all in the fully chaotic region:

1. Arnold’s cat map:

$$\left\{\begin{matrix} x_{n+1}=x_{n}+y_{n} (\text{Mod }1) \\ y_{n+1}=x_{n}+2y_{n} (\text{Mod }1) \end{matrix}\right.$$

1. Chirikov standard map:

$$\left\{\begin{matrix} x_{n+1}=x_{n}+k\sin(y_{n}) (\text{Mod 2}\pi) \\ y_{n+1}=x_{n+1}+y_{n} (\text{Mod 2}\pi) \end{matrix}\right.$$

with $k=0.2$.

1. Gingerbreadman map:

$$\left\{\begin{matrix} x_{n+1}=1-y_{n}+\left|x_{n}\right| \\ y_{n+1}=x_{n} \end{matrix}\right.$$

Ornstein-Uhlenbeck process is defined as[1-4]

${dx}_{t} =\theta\left( \mu-x_{t} \right)dt+\sigma dW_{t}$

with $\theta=1，\mu=1.2，\sigma=0.3 \mathrm{and} W_{t}$ represents the Wiener process.

Lorenz system with a stochastic forcing is introduced as:

$\left\{ \begin{matrix} \dot{x}=a \left( y-x \right)+A*\mathrm{randn}(1,1) \\ \dot{y}=bx-y-xz+A*\mathrm{randn}(1,1) \\ \dot{z}=xy-cz+A*\mathrm{randn}(1,1) \end{matrix} \right.$

where *a=10, b=25*, and *c=8/3*; $randn(1,1)$ represents random data. Here *A* is taken as the ratio of standard deviation between the additive noise and the original chaotic system without adding noise.

High-dimensional chaotic Mackey-Glass system [5-7]

$\dot{x}=\frac{ax_{\tau}}{\left( 1+{x_{\tau}}^{n} \right)}-bx;x_{\tau}=x(t-\tau)$

with $a=0.2, b=0.1, n=10, \tau=10$.

**Figure A. The first digit distribution at different scales for four stochastic time series.** FGN ($\beta=0.2$), FGN ($\beta=0.4$), FBM ($\beta=2.2$) and K-noise ($k=3.0$**).** We can see that though the first digit distribution changes as scale changes, the fundamental feature is still heavily skewed toward the smaller digits and the ED value do not change much.

**Figure B. The first digit distribution at different scales for four noninversative chaotic maps.** Skew tent map, linear congruential generator, Schuster and and Gauss map-$x$.

**Figure C. The first digit distribution at different scales for four dissipative chaotic maps.** Ideka map, Lozi map-$y$, Tinkerbell map-$x$ and Kaplan-Yorke map-$y$.

**Figure D. The first digit distribution at different scales for four conservative chaotic maps.** Arnold cat map, Gingerbreadman map-$x$, Lorenz map-$x$ and Chirikov standard map-$x$**.**

**Figure E. The changing** $ED(s)$ **with scale factor s for three kinds of stochastic processes.**

**Figure F. The results of** $ED(s)$ **for five noninversative chaotic maps comparing with those from stochastic processes.**

**Figure G. The results of** $ED(s)$ **for nine dissipative chaotic maps comparing with those from stochastic processes.**

**Figure H. The results of** $ED(s)$ **for four conservative chaotic maps comparing with those from stochastic processes.**

**Figure I. The results of** $\Delta ED(L)$**for five noninversative chaotic maps comparing with those from stochastic processes.**

**Figure J. The results of** $\Delta ED(L)$ **for nine dissipative chaotic maps comparing with those from stochastic processes.**

**Figure K. The results offor four conservative chaotic maps comparing with those from stochastic processes.**

**Figure L. The comparative results for different quantifiers.** The Euclidean distance versus scale factor, the Wootters’s distance versus scale factor, the range of Euclidean distance versus scale range, and the range of Wootters’s distance versus scale range. Similar with Euclidean distance, the Wootters’s distance (WD) is another quantifier to calculate that to what extent the observed probability differs from the theoretical probability. It is defined as $WD(s)=\cos^{-1}\{\sum_{d=1}^{9} [P_{B}(d)]^{2}\bullet[P_{s}(d)]^{2}$, where $P_{B}(d)$ is the probability based on Benford’s law, $P_{s}(d)$ is the observed first digit distribution at scale $s$. The results indicate our method is robust with respect to different quantifiers, even other distances in probability space are chosen.

**Figure M. The** $\Delta ED(L=10)$ **versus different data lengths for chosen CSs and SPs.** It indicates this quantifier works well under small data length with a minimum data length of 4,000 points.

**Figure N.** **The results for Ornstein-Uhlenbeck process: (a) $\boldsymbol{E}\boldsymbol{D}$, (b)** $\boldsymbol{\Delta E}\boldsymbol{D}$

**Figure O.** $\boldsymbol{\Delta E}\boldsymbol{D}$ **for Mackey-Glass system.**

**Figure P.** $\boldsymbol{\Delta E}\boldsymbol{D}$ **for Lorenz system with colored noise FGN ($\boldsymbol{\beta}\mathbf{=0.6}$)**

**Figure Q.** **Results of $\boldsymbol{\Delta E}\boldsymbol{D}$ for Lorenz system with stochastic forcing**

**References:**

[1] Ricciardi LM, Sato S. First-passage-time density and moments of the Ornstein-Uhlenbeck process. Journal Of Applied Probability. 1988:43-57.

[2] Alili L, Patie P, Pedersen JL. Representations of the first hitting time density of an ornstein-uhlenbeck process 1. Stochastic Models. 2005;21:967-80.

[3] Gillespie DT. Exact numerical simulation of the Ornstein-Uhlenbeck process and its integral. Physical Review E. 1996;54:2084.

[4] Vannitsem S. Bias correction and post-processing under climate change. Nonlinear Processes In Geophysics. 2011;18:911-24.

[5] Shahverdiev EM, Nuriev RA, Hashimov RH, Shore KA. Chaos synchronization between the Mackey–Glass systems with multiple time delays. Chaos, Solitons & Fractals. 2006;29:854-61.

[6] Namajūnas A, Pyragas K, Tamaševičius A. Stabilization of an unstable steady state in a Mackey-Glass system. Physics Letters A. 1995;204:255-62.

[7] Farmer JD, Sidorowich JJ. Predicting chaotic time series. Physical Review Letters. 1987;59:845.
